# Enterohemorrhagic *Escherichia coli* (EHEC) disrupts intestinal barrier integrity in translational canine stem cell-derived monolayers

**DOI:** 10.1128/spectrum.00961-24

**Published:** 2024-08-20

**Authors:** Itsuma Nagao, Minae Kawasaki, Takashi Goyama, Hyun Jung Kim, Douglas R. Call, Yoko M. Ambrosini

**Affiliations:** 1 Department of Veterinary Clinical Sciences, College of Veterinary Medicine, Washington State University, Pullman, Washington, USA; 2 Department of Veterinary Internal Medicine, Graduate School of Agricultural and Life Sciences, The University of Tokyo, Tokyo, Japan; 3 Department of Inflammation and Immunity, Lerner Research Institute, Cleveland Clinic, Cleveland, Ohio, USA; 4 Paul G. Allen School for Global Health, Washington State University, Pullman, Washington, USA; Ludwig-Maximilians-Universitat Munchen Pettenkofer Institute, München, Germany

**Keywords:** dog, EHEC, organoid, colon

## Abstract

This study addresses the gap in translatable *in vitro* models for investigating Enterohemorrhagic *E. coli* (EHEC) infections, particularly relevant to both canine and human health. EHEC is known to induce acute colitis in dogs, leading to symptoms like hemorrhagic diarrhea and hemolytic uremic syndrome, similar to those observed in humans. However, understanding the pathophysiology and developing treatment strategies have been challenging due to the lack of effective models that replicate the clinical disease caused by EHEC in both species. Our approach involved the development of colonoid-derived monolayers using intestinal tissues from healthy, client-owned dogs. These monolayers were exposed to EHEC, and the impact of EHEC was assessed through several techniques, including trans-epithelial electrical resistance (TEER) measurement, immunofluorescence staining for junction proteins and mucus, and scanning electron microscopy for morphological analysis. Modified culture with saline, which was intended to prevent bacterial overgrowth, maintained barrier integrity and cell differentiation. EHEC infection led to significant decreases in TEER and ZO-1 expression, but not in E-cadherin levels or mucus production. In addition, EHEC elicited a notable increase in tumor necrosis factor-alpha production, highlighting its distinct impact on canine intestinal epithelial cells compared to non-pathogenic *E. coli*. These findings closely replicate *in vivo* observations in dogs and humans with EHEC enteropathy, validating the canine colonoid-derived monolayer system as a translational model to study host-pathogen interactions in EHEC and potentially other clinically significant enteric pathogens.

**IMPORTANCE:**

This study develops a new model to better understand Enterohemorrhagic *E. coli* (EHEC) infections, a serious bacterial disease affecting both dogs and humans, characterized by symptoms such as hemorrhagic diarrhea and hemolytic uremic syndrome. Traditional research models have fallen short of mimicking how this disease manifests in patients. Our research used intestinal tissues from healthy dogs to create layers of cells, known as colonoid-derived monolayers, which we then exposed to EHEC. We assessed the damage caused by the bacteria using several techniques, observing significant changes similar to those seen in actual cases of the disease. The model proved effective in replicating the interaction between the host and the pathogen, marking an important step toward understanding EHEC’s effects and developing treatments. This canine colonoid-derived monolayer system not only bridges a crucial gap in current research but also offers a promising platform for studying other enteric pathogens affecting both canine and human health.

## INTRODUCTION

Human acute colitis is frequently attributed to food-borne infections caused by Enterohemorrhagic *Escherichia coli* (EHEC) that produce Shiga toxin (Stx). This toxin induces cytotoxic effects on adjacent endothelial cells, resulting in symptoms such as bloody diarrhea and hemolytic uremic syndrome (HUS) (1). While studying EHEC, researchers have encountered challenges in modeling the disease. Mouse models, for example, failed to replicate the expected effects of mucosal colonization and intestinal damage (2).

Although the pathogenicity of EHEC in dogs is relatively mild (3), acute colitis due to EHEC infection, including hemorrhagic diarrhea and HUS similar to humans, has been demonstrated in experimentally infected dogs or critically ill dogs (4, 5). Because of this similarity, there is a growing interest in using dogs as *in vivo* models for human EHEC infections and as sources of cells and tissues for *in vitro* models.

Recent advancement of organoid technology offers improved *in vitro* models compared to traditional cell culture systems (6, 7). Organoid models include three-dimensional intestinal organoids. Organoids derived from adult stem cells closely mimic the *in vivo* microenvironment and maintain multi-cell lineage cultures (7). Two-dimensional (2D) monolayers can also be derived from organoids (8). These 2D monolayers offer a stable and accessible luminal interface to examine nutritional effects, drug permeability, and host-pathogen interactions. In addition, sophisticated microfluidic intestine-on-a-chip systems have been established to study changes in the intestinal microenvironment and host responses with more physiologically complex *in vitro* models (9, 10).

While there is a wealth of literature on murine and human intestinal organoids and associated technologies, research on canine equivalents is still emerging (11). Canine models are gaining prominence due to their relevance as a spontaneous, large animal model for various human intestinal diseases, including EHEC infection. Therefore, the present study aimed to establish a comparative *in vitro* canine 2D monolayer model to assess the effects of EHEC infection, utilizing canine colonoid-derived monolayers and a strain of non-pathogenic *E. coli* as infection control. Our hypotheses are based on the expected biological responses observed in similar models and clinical cases. Specifically, we anticipate that EHEC adherence to canine monolayers will be lower than in human studies due to a reduced number of goblet cells in our model, potentially reflecting the milder clinical symptoms seen in dogs. We also hypothesize that EHEC infection will compromise barrier integrity through the downregulation of tight junction proteins and reduction of trans-epithelial electrical resistance (TEER), differing significantly from non-infected and non-pathogenic *E. coli*-infected controls. Furthermore, as no studies currently explore mucus production in dogs post-EHEC infection, we aim to bridge this knowledge gap, predicting less pronounced changes compared to other species. Finally, we expect an increase in the inflammatory cytokines interleukin (IL)-8 and tumor necrosis factor-alpha (TNF-α), indicating a robust immune response in the canine model. This comprehensive approach will enhance our understanding of EHEC’s impact on canine gut health and validate our *in vitro* model.

## RESULTS

### Multilineage cell differentiation and intestinal barrier integrity of monolayers were maintained with saline

**Fig 1:**
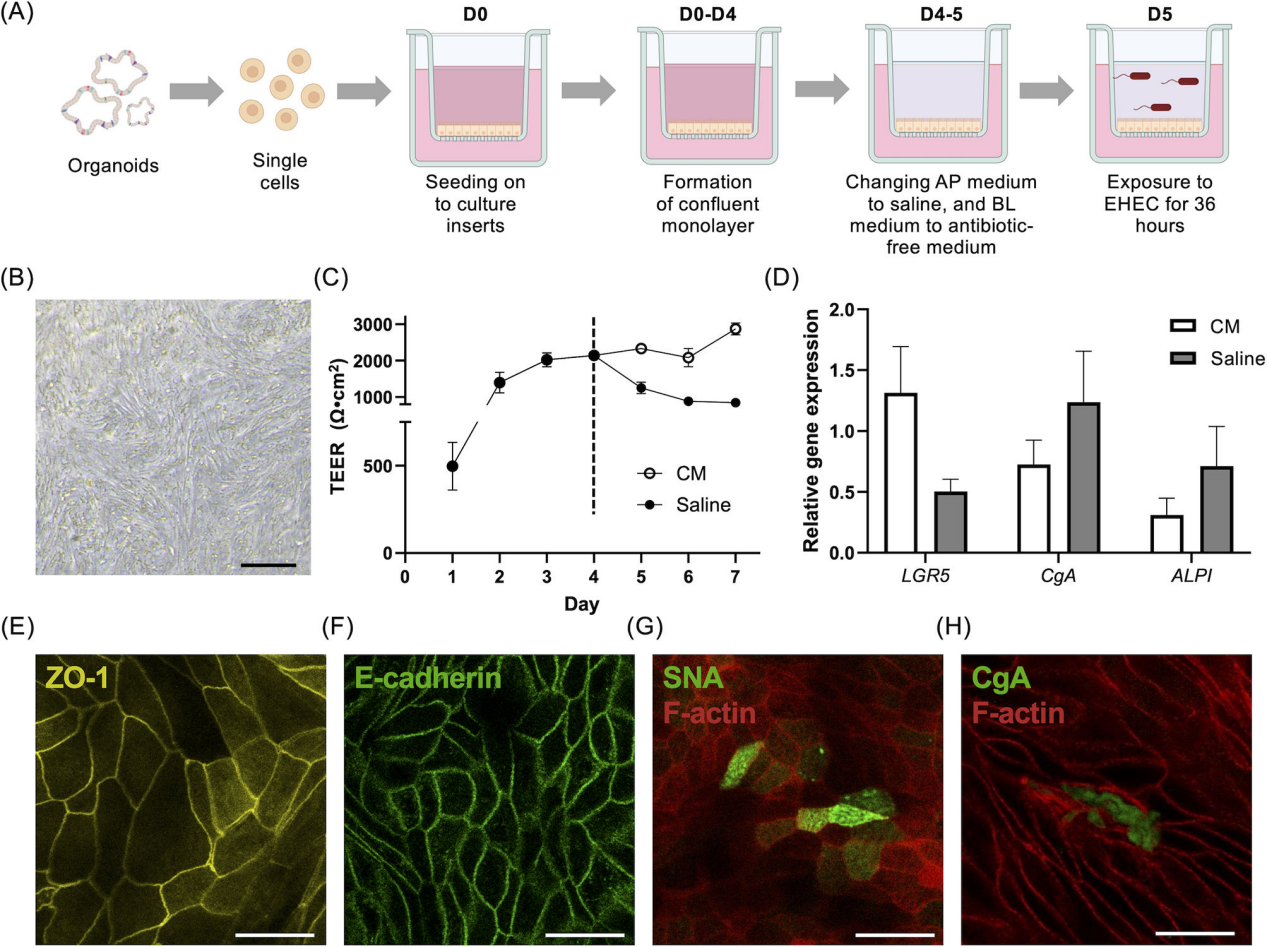
Modification of canine colonoid-derived monolayers for investigating EHEC infection. (**A**) A schematic diagram of the experiment. Colonoid-derived monolayers were derived from canine colonoids (**D0-D4**), and the organoid culture medium was replaced with saline in the apical chamber to avoid overgrowth of bacteria and antimicrobial-free organoid culture medium in the basolateral chamber once the monolayers reached a stable state at day 4 (**D4**). Then, co-culture with EHEC was conducted on day 5 (**D5**). This schematic was created with BioRender.com. (**B**) Representative phase-contrast image of colonoid-derived monolayers after 3 days of culture using saline. Scale bar = 100 µm. (**C**) Changes in TEER value over time. Colonoid-derived monolayer was cultured using CM for the first 4 days, and after the measurement of TEER on day 4, the culture medium was switched into saline (black circle) or maintained as CM (white circle). The dashed line indicates the timing of the switch to the two types of culture media. (**D**) After 3 days of culture in saline, the expression levels of differentiated cell marker genes [*leucine-rich repeat containing G protein-coupled receptor 5* (*LGR5*), *Chromogranin A* (*CgA*), and *Intestinal Alkaline Phosphatase* (*ALPI*)] in the colonoid-derived monolayers were quantified by RT-qPCR. CM, a stem cell maintenance medium, was used for comparison. The error bars represent the standard error of the mean. (**E–H**) Representative fluorescence images of colonoid-derived monolayers after 3 days of culture using saline. Immunofluorescence staining on canine colonoid-derived monolayers confirms the expression of tight junction protein, ZO-1 (Yellow: E) and E-cadherin (Green: F). The population of goblet cells (SNA: Green: G) and enteroendocrine cells [Chromogranin A (CgA): Green: H] was also visualized using immunofluorescence staining with a counterstaining, F-actin (Red). Scale bar = 25 µm.

In this study, we modified the apical medium 24 hours before infection by switching from the standard organoid culture medium to a nutrient-free saline solution. This adjustment aimed to prevent bacterial overgrowth in the medium, as previously reported (12) (Fig. 1A). Initially, we assessed whether the colonoid-derived monolayers, which had been cultured in the organoid culture medium for 4 days, could be sustained in saline for 3 days, aligning with the desired duration of co-culture with bacteria. During the 3-day saline culture period, the colonoid-derived monolayers maintained their confluency (Fig. 1B). Stable TEER values were maintained throughout the monolayer culture, even after changing the apical medium from complete medium (CM) to saline. It is important to note that although a decrease in TEER measurements was observed upon switching to saline (Fig. 1C), the values eventually stabilized around 881.8 Ω•cm^2^. In addition, to verify the multi-lineage characteristics of the monolayers, we utilized quantitative polymerase chain reaction (qPCR). This technique was instrumental in determining whether there was a variation in the composition of differentiated cells between two groups: one being colonoid-derived monolayers cultured in nutrient-free saline, and the other in a standard organoid culture medium. Our analysis revealed no significant differences between the two groups for the expression levels of stem cell marker gene *leucine-rich repeat containing G protein-coupled receptor 5* (*LGR5*), the enteroendocrine cell marker gene *Chromogranin A* (*CgA*), and the enterocyte marker gene *Intestinal Alkaline Phosphatase* (*ALPI*) (Fig. 1D). In addition, the formation of functional tight junctions was confirmed using immunocytochemistry (Fig. 1E and F). To further confirm the presence of differentiated goblet cells, we conducted staining using *Sambucus nigra* Agglutinin (SNA), which is a lectin that identifies sialic acid bound to galactose in mucin (13). This staining revealed the presence of SNA-positive cells on the colonoid-derived monolayers (Fig. 1G). To further confirm the presence of differentiated enteroendocrine cells, we performed staining with CgA, which revealed CgA-positive cells in the colonoid-derived monolayers (Fig. 1H; Fig. S1).

### Enhanced visualization of mucus ultrastructure with modified scanning electron microscopy fixation protocol

**Fig 2:**
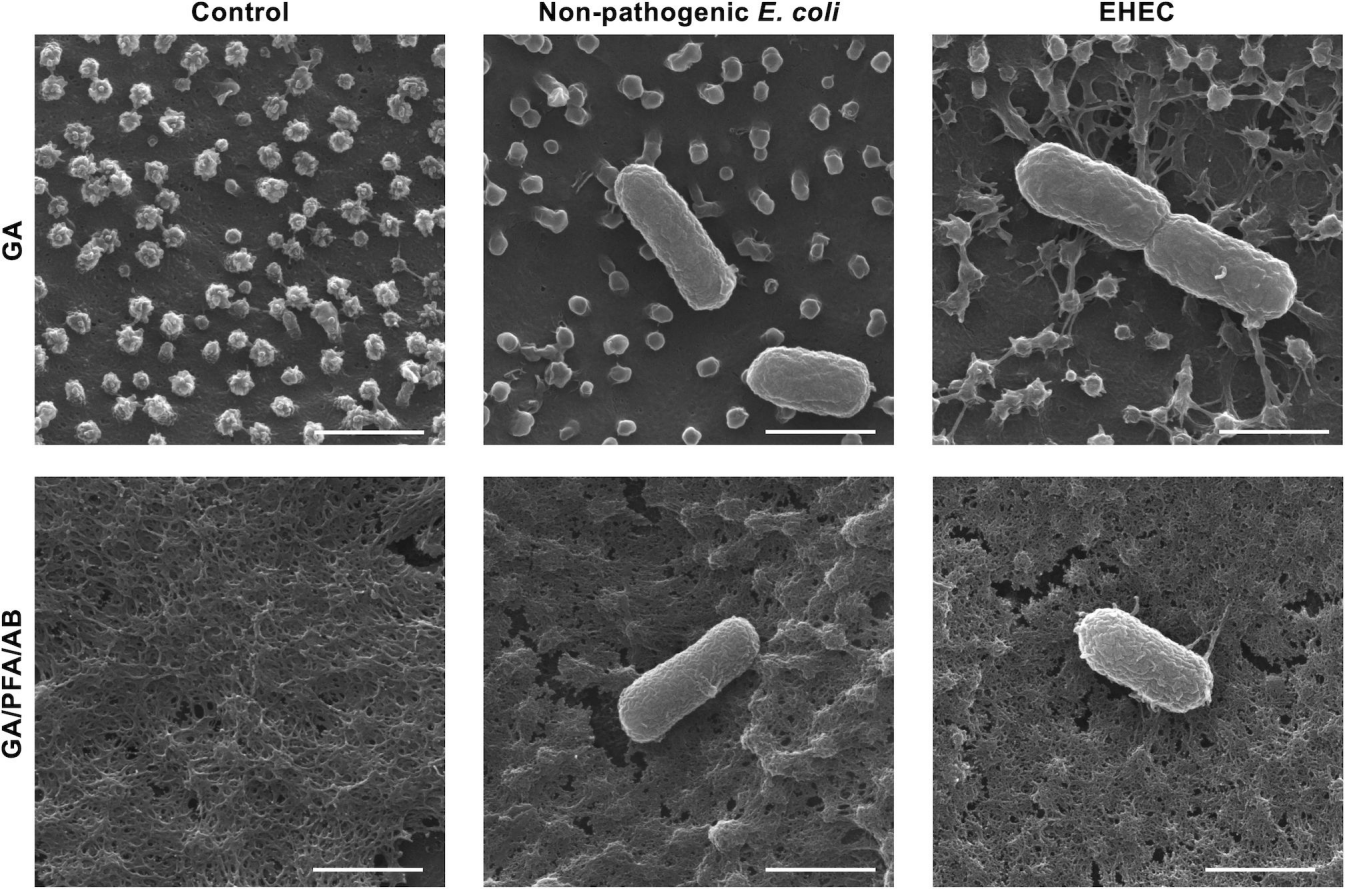
EHEC and non-pathogenic *E. coli* attachment on microvilli and mucus ultrastructure in canine colonoid-derived monolayers. After 36 hours of co-culture, the cells were fixed and their surfaces were observed with a scanning electron microscope. Representative images of uninfected control, non-pathogenic *E. coli*, and EHEC are shown on the left, middle, and right, respectively. In the top images, GA fixation resulted in minimal mucus preservation, providing a clear view of the apical surface cellular structures (**A**). By contrast, the bottom images show that GA/PFA/AB fixation retained a more substantial mucus layer, facilitating the visualization of the mucus ultrastructure, which exhibited a uniform, net-like 3D structure across the monolayers (**B**). Scale bar: 1 µm.

Two fixation methods were compared for their effectiveness in preserving and visualizing the mucus ultrastructure *via* scanning electron microscopy (SEM). The methods included using glutaraldehyde (GA) alone and a combination of GA, paraformaldehyde, and alcian blue (GA/PFA/AB). Analysis of control monolayers that were not exposed to bacteria revealed that fixation with GA alone minimally preserved the mucus layer on the apical epithelial surface, which enhanced the clarity of underlying cellular features such as microvilli (Fig. 2A). Conversely, the GA/PFA/AB fixation method retained a more uniform mucus layer, thereby improving the visualization of the mucus ultrastructure across the monolayer’s apical surface (Fig. 2B) (13).

### Establishment of EHEC co-culture on canine colonoid-derived monolayers

**Fig 3:**
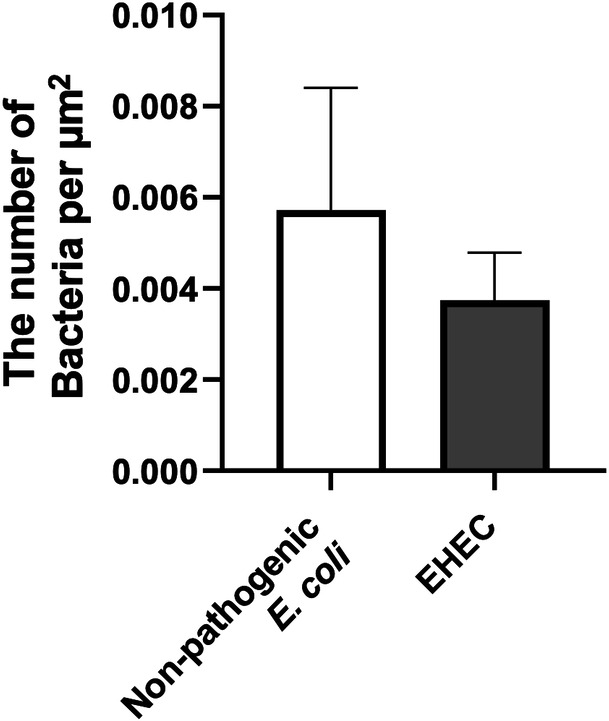
Quantification of the number of bacteria adhering to the apical surface of the monolayers. Quantification of adhering bacteria on the apical surface of the monolayers observed on SEM images fixed with 2.5% glutaraldehyde in 0.1 M sodium cacodylate buffer. Ten independent fields of view at 1,542× magnification from three independent experiments using three biological replicates were analyzed. The error bars represent the standard error of the mean.

Next, EHEC isolated from cattle was co-cultured with the colonoid-derived monolayers. Because clinical isolates of EHEC from dogs were not available, a clinical isolate from cattle was used. In addition, a non-pathogenic *E. coli* strain, MC4100, was used as an infection control strain for *E. coli* infection (14). At 36 hours post-infection, the epithelial surface of all uninfected control cultures, as well as those infected with non-pathogenic *E. coli* or EHEC, displayed uniformly extended microvilli (Fig. 2A). Although bacteria adhered to the epithelial cells in both the non-pathogenic *E. coli* and EHEC cultures, the patterns of attachment differed significantly. Non-pathogenic *E. coli* was found adhering to the cell surface but not specifically to the microvilli. By contrast, EHEC not only adhered to the cell surface but also appeared to congregate around and bind strongly to the microvilli, with evidence of microvilli bending toward the EHEC bacteria (Fig. 2A). In GA/PFA/GA fixation, which preserves mucus, there were no clear differences in the morphology and quantity of mucus in the three groups: uninfected control, non-pathogenic *E. coli*, and EHEC (Fig. 2B). Quantitative analysis suggested a trend of higher bacterial adherence on the apical surface of monolayers in the non-pathogenic *E. coli* group compared to the EHEC group, although this difference did not reach statistical significance (Fig. 3).

**Fig 4:**
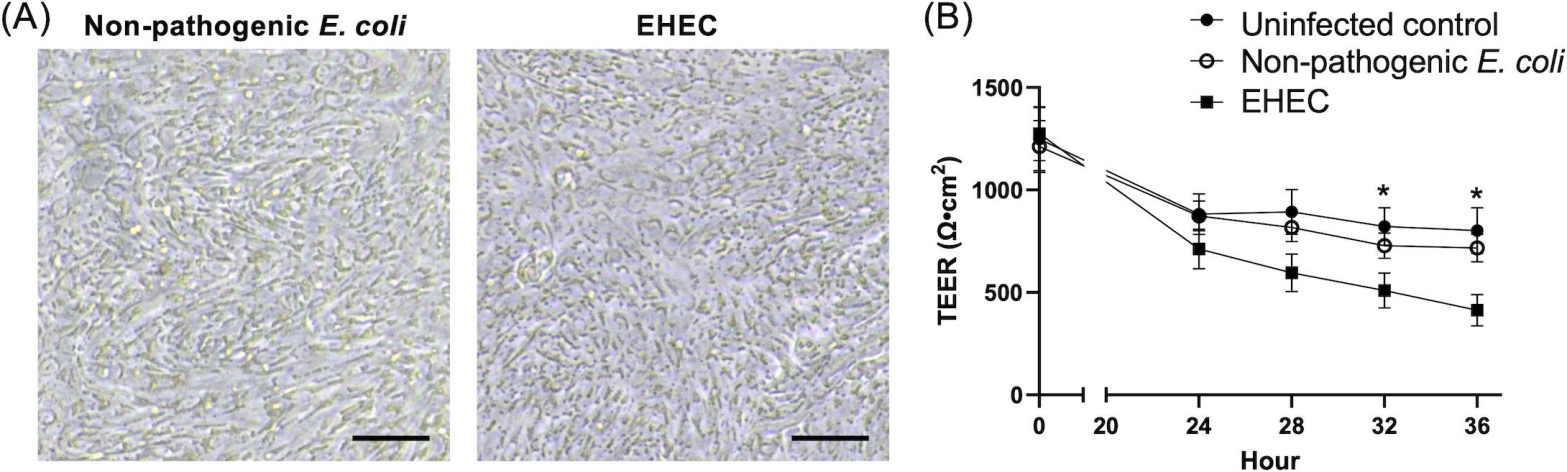
Evaluation of epithelial barrier integrity in canine colonoid-derived monolayers co-cultured with bacteria. (**A**) Representative phase-contrast image of colonoid-derived monolayers after 36 hours of co-culture with bacteria. Scale bar = 100 µm. (**B**) Changes in TEER value since the initiation of co-culture. TEER values were measured up to 36 hours after the start of co-culture. Black circles indicate the uninfected control group, white circles indicate the non-pathogenic *E. coli* group, and black squares indicate the EHEC group. Statistical comparisons were made at each time point, with significantly lower TEER in the EHEC group compared to the uninfected control and non-pathogenic *E.coli* groups at 32 and 36 hours. *P < 0.05.

Phase-contrast microscopy indicated no major differences in appearance between cultures treated with non-pathogenic *E. coli* or EHEC, with both maintaining confluent monolayers (Fig. 4A). TEER values decreased from the start of culture for all cultures for the first 24 hours but were maintained in the uninfected control and non-pathogenic *E. coli* cultures thereafter. By contrast, the TEER values for the EHEC-infected monolayers continued to decline, showing a significant decrease compared to the non-pathogenic *E. coli* infection at 32 hours and 36 hours post-coculture (Fig. 4B) (*P* = 0.03 and *P* = 0.01, respectively).

### Expression of ZO-1, but not E-cadherin, decreased following EHEC co-culture

**Fig 5:**
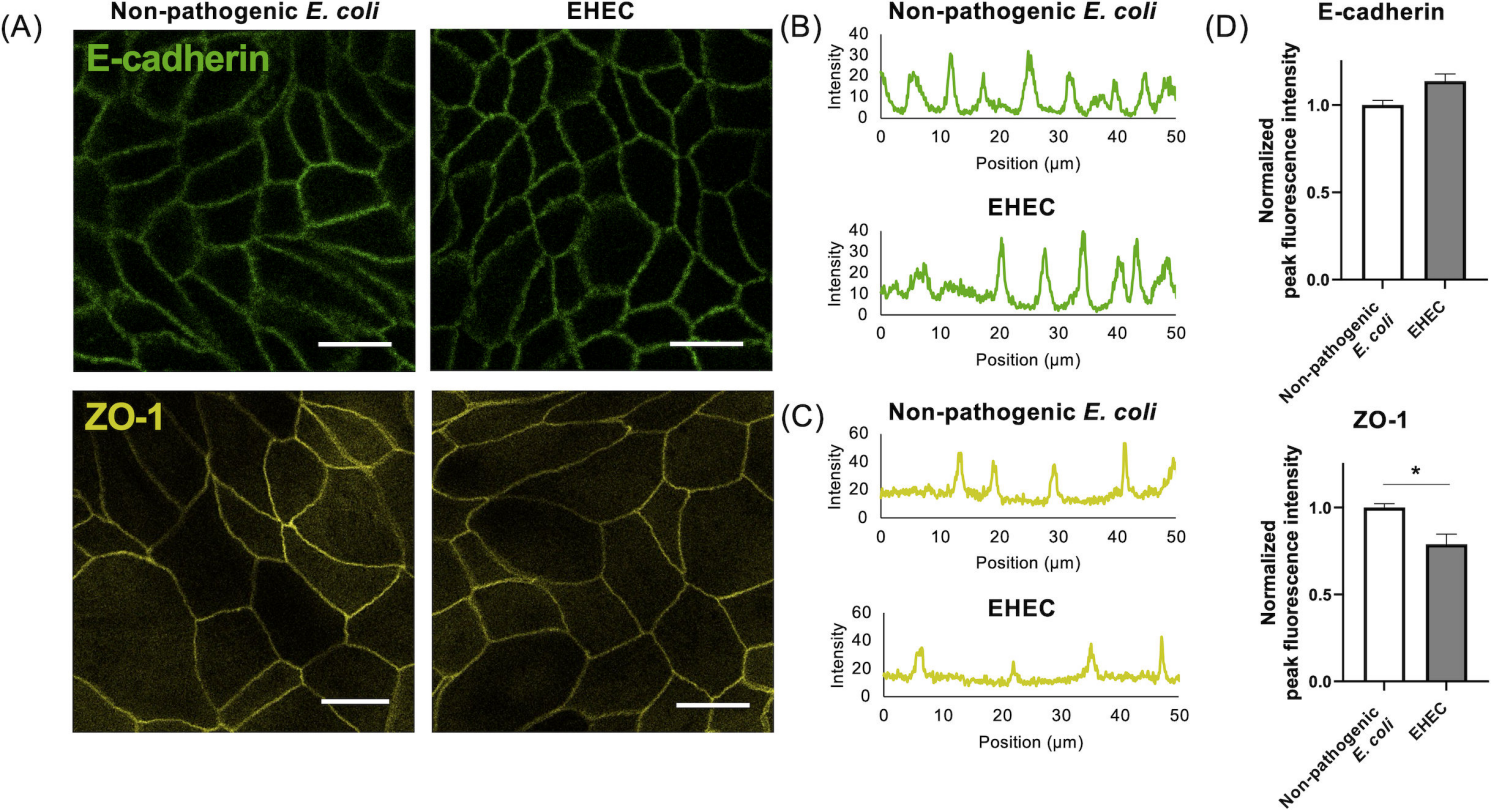
Tight junction proteins in canine colonoid-derived monolayers after bacterial co-culture. (**A**) Representative fluorescence image of tight junction proteins, E-cadherin (Green) and ZO-1 (Yellow), in colonoid-derived monolayers after 36 hours of co-culture with bacteria. Scale bar = 25 µm. (**B**) Representative line scan image of E-cadherin. The upper graph shows the non-pathogenic *E. coli* group and the lower graph shows the EHEC group. (**C**) Representative line scan image of ZO-1. The upper graph shows the non-pathogenic *E. coli* group and the lower graph shows the EHEC group. (**D**) Comparison of the peak intensity in the line scan. To evaluate intercellular specific fluorescence intensity, a comparison of the intensity of each peak on the line scan was performed. This measurement was performed using three biological replicates with three technical replicates. For all samples, images were obtained in five randomly selected fields of view, and a line scan was performed on each image. In each biological replicate, the fluorescence intensity was normalized by the mean of the peak fluorescence intensity in non-pathogenic *E. coli*. White bars indicate the non-pathogenic *E. coli* group and gray bars indicate the EHEC group. The error bars represent the standard error of the mean. *P < 0.05.

As previously reported, EHEC infection is known to downregulate tight junction protein expression and disrupt the gut barrier (15–17). We evaluated the expression levels of tight junction proteins, namely ZO-1 and E-cadherin, in canine colonoid-derived monolayers using immunofluorescence staining after 36 hours of co-culture (Fig. 5A). Fluorescence images of both non-pathogenic *E. coli* and EHEC cultures showed distinct tight junctions in the intercellular regions with no obvious disruption of tight junctions. To determine whether there were differences in the intensity of tight junction expression, we performed line scans using fluorescence images to extract intercellular-specific fluorescence intensities and compared these intensities between the two groups (Fig. 5B and C). While the expression of E-cadherin was not different between the two groups (*P* = 0.06), ZO-1 decreased significantly for EHEC-exposed monolayers (*P* = 0.02) (Fig. 5D).

### Mucus production in the canine colonoid-derived monolayers was not altered by EHEC infection

**Fig 6:**
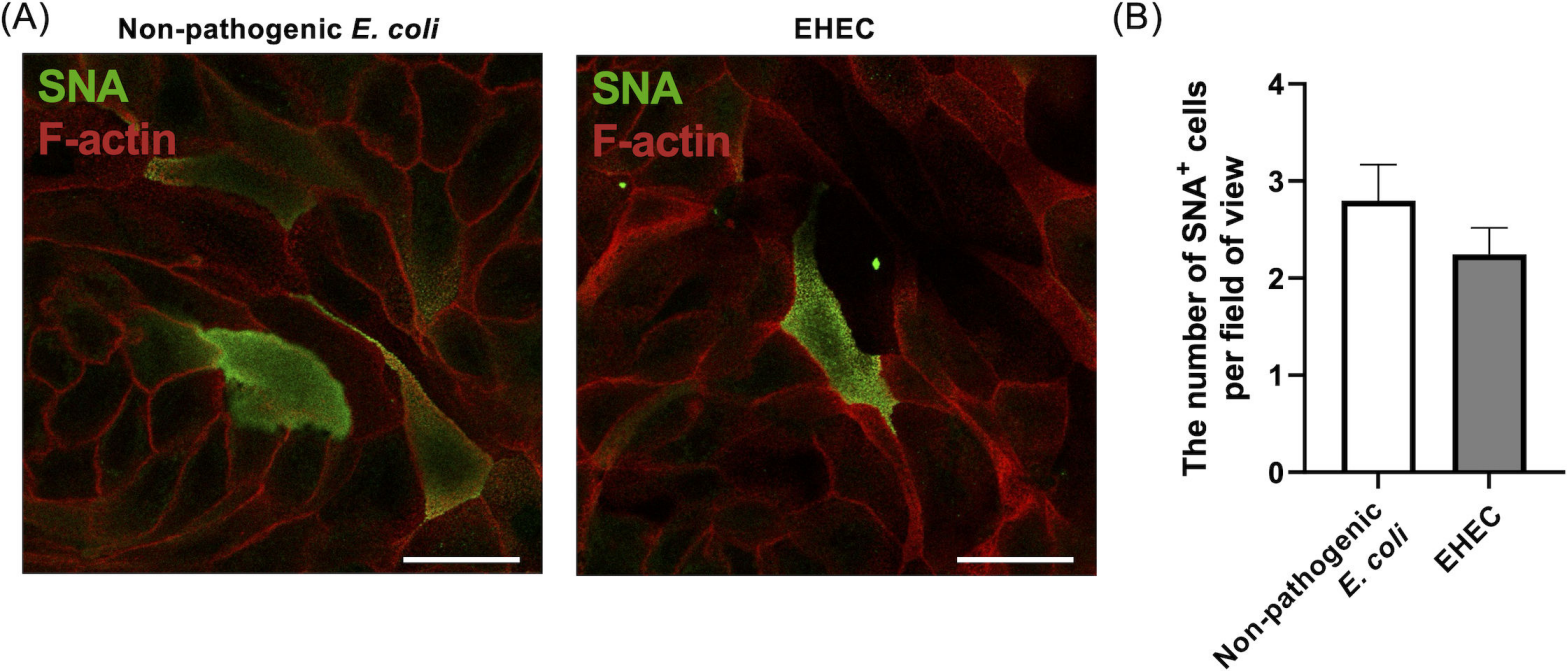
Evaluation of mucus production in canine colonoid-derived monolayers after bacterial co-culture. (**A**) Representative fluorescence image of SNA staining. SNA is a lectin that binds to sialic acid in the sugar chains of mucus, which could be interpreted to mean that SNA-positive cells are goblet cells. Scale bar = 25 µm. (**B**) Quantification of SNA-positive cells. To calculate the percentage of SNA-positive cells in each group, the number of SNA-positive cells per field of view under 63× magnification was counted. This quantification was performed in five randomly selected fields of view in each sample with three technical replicates using three biological replicates. White bars indicate the non-pathogenic *E. coli* group and gray bars indicate the EHEC group. The error bars represent the standard error of the mean. *P < 0.05.

We assessed the mucus production of canine colonoid-derived monolayers after the co-culture with non-pathogenic *E. coli* or EHEC because changes in mucus-producing function have been reported in mice and humans (17, 18). After 36 hours of co-culture with the bacteria, SNA staining was performed and the number of SNA-positive cells per high magnification field of view was calculated. The number of SNA-positive cells per field of view was 2.8 ± 0.3 cells with the non-pathogenic *E. coli* co-culture and 2.4 ± 0.3 cells with the EHEC co-culture (*P* = 0.27) (Fig. 6).

### Inflammatory cytokine production was elicited by EHEC infection

**Fig 7:**
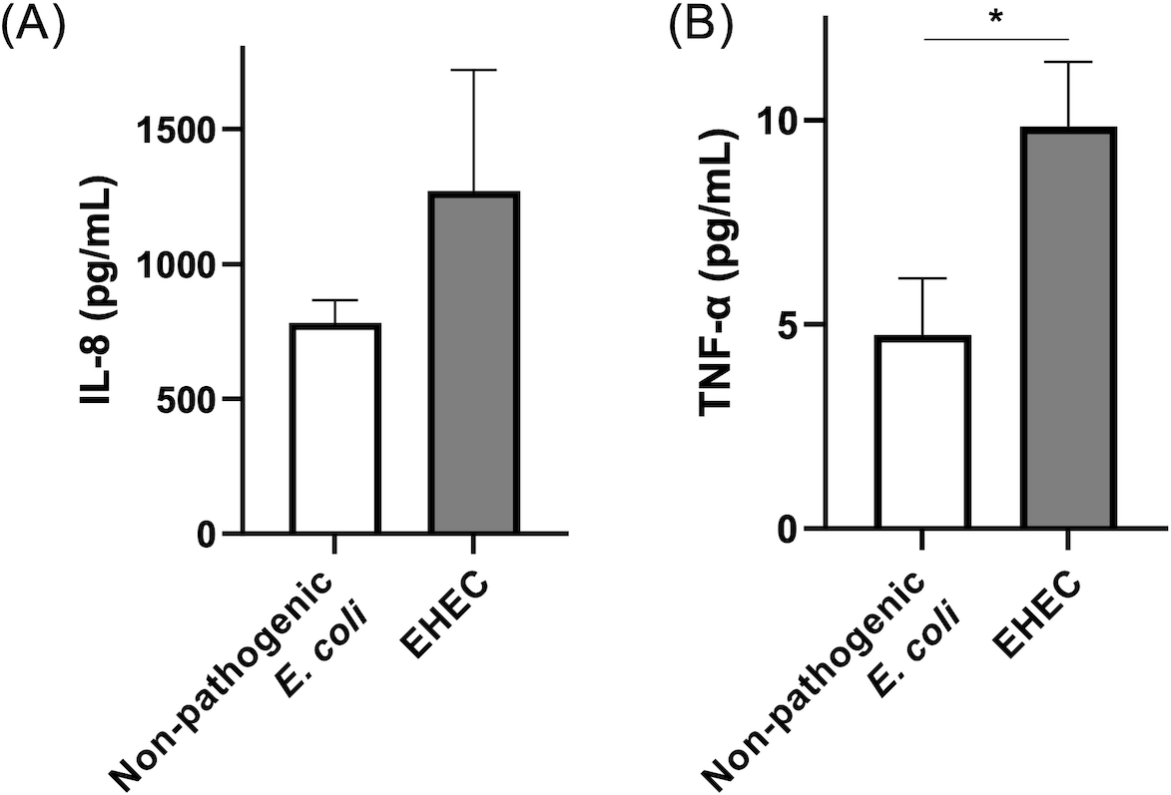
Inflammatory cytokine production in canine colonoid-derived monolayers co-cultured with bacteria. Concentration of inflammatory cytokine, IL-8 (**A**) and TNF-α (**B**) was measured using the basolateral culture medium after 36 hours of co-culture by ELISA. White bars indicate the non-pathogenic *E. coli* group and gray bars indicate the EHEC group. The error bars represent the standard error of the mean. *P < 0.05.

We measured the concentration of IL-8 and TNF-α in the medium after 36 hours of co-culture with bacteria because these cytokines are upregulated in the intestinal tract after EHEC infection (19–21). In accordance with previous studies, these measurements were performed by ELISA using basolateral medium (21). The concentration of IL-8 (782.7 ± 84.1 pg/mL) from exposure to non-pathogenic *E. coli* was similar to the concentration for the EHEC-exposed monolayers (1,271.0 ± 448.9 pg/mL) (*P* = 0.54, Fig. 7A). The concentration of TNF-α was significantly higher with EHEC co-culture (9.8 ± 1.6 pg/mL) compared with non-pathogenic *E. coli* co-culture (4.7 ± 1.4 pg/mL) (*P* = 0.03, Fig. 7B).

## DISCUSSION

This study introduces a novel canine colonoid-derived monolayer model for *in vitro* studies of EHEC infection. We have found that incorporating a saline-only incubation step effectively limits bacterial overgrowth without causing undesirable artifacts to the monolayers. In addition, results from the non-pathogenic *E. coli* co-culture provide a useful contrast for evaluating specific effects of EHEC infection on canine intestinal epithelial cells.

Cattle are the primary reservoirs for EHEC and the transmission of this bacterium to humans primarily occurs through the consumption of contaminated food products derived from cattle, the use of cattle manure as fertilizer, and water supplies contaminated by runoff from cattle farms (22). In addition, dogs consuming raw food diets are increasingly at risk of being contaminated with enteric pathogens, including antibiotic-resistant strains of *E. coli* and *Salmonella enterica* (23). In our study, we utilized an EHEC isolate sourced from cattle feces to more accurately mirror the natural infection route in dogs. While concerns may arise regarding differences between canine- and bovine-sourced isolates, evidence of host specificity among various *E. coli* lineages does not extend to *E. coli* serovar O157:H7 (22). Therefore, we anticipate that the outcomes would be similar regardless of the isolate’s origin. This approach offers a realistic representation of potential EHEC exposure in the canine environment.

In SEM analyses of epithelial monolayers derived from canine colonoids, the adherence of EHEC to the epithelium was observed. The GA/PFA/AB fixation method was found to preserve mucus ultrastructure more effectively compared to the GA-only method. However, the GA-only method provided a clearer visualization of interactions between microvilli and *E. coli* microbes. Non-pathogenic *E. coli* was observed adhering to the cell surface but did not specifically bind to microvilli. Conversely, EHEC not only adhered to the cell surface but also congregated around and strongly bound to microvilli, with microvilli bending toward the bacteria. This behavior is likely due to the action of effector proteins secreted by EHEC *via* the Type III secretion system (T3SS), facilitating bacterial entry into the host cells (24).

The number of EHEC attached to the monolayer surface was lower than that of non-pathogenic *E.coli* (Fig. 3). However, the number of bacteria that adhered was relatively low. Previous research indicates that EHEC colonizes the epithelial surface by utilizing its flagella to attach to the mucus produced by intestinal goblet cells (25). This highlights the crucial role of intestinal mucus in EHEC infections. The monolayers in our study, however, were cultured in a stem cell maintenance medium, which resulted in a high population of stem cells and a markedly low population of goblet cells (26). This condition was anticipated to reduce the number of adhering bacteria. Supporting this, a study involving human intestinal organoids demonstrated that EHEC adherence was significantly higher (by 10-fold) in monolayers with increased mucus secretion, achieved through induced differentiation, compared to those cultured in a stem cell maintenance medium (18). This suggests the importance of developing methods to differentiate canine organoid-derived monolayers for more effective studies on EHEC infection in the future.

In our current model, membrane integrity was evaluated using TEER measurements. Following the switch from CM to saline, a slight decrease in TEER values was observed (Fig. 1C), which could be attributed to physiological adjustments in the cells due to the medium change (23). Importantly, these values are stabilized at approximately 881.8 Ω•cm² (Fig. 1C), indicating consistent membrane integrity. This observation aligns with findings from previous studies that demonstrated stable membrane integrity under similar conditions (8, 23). The implications of these TEER findings reach beyond our *in vitro* model and have significant real-world relevance. The Centers for Disease Control and Prevention (CDC) estimates that foodborne infections caused by EHEC result in approximately 63,000 cases annually in the United States, leading to over 2,100 hospitalizations and fatalities (27). The economic impact of these illnesses, considering medical costs, loss of life, and reduced productivity, is estimated at $405 million each year (28). Focusing specifically on severe EHEC infections, about 10% of patients with infections from Shiga toxin-producing *E. coli* (STEC) may develop HUS, with a mortality rate of 3%–5% (29). In canine subjects, severe EHEC infections leading to HUS have been documented, though predominantly in experimental settings or dogs with compromised immunity (4, 5). Notably, in our study, the impact of EHEC on TEER, that is, a modest reduction by about 500 Ω•cm², mirrors the relatively mild alterations observed in naturally occurring EHEC infections in dogs.

Various *in vitro* studies have reported a reduction in colon mucus in cell culture (18, 26), yet *in vivo* information remains scarce. Research has shown that EHEC can gain a competitive edge by utilizing mucus-derived sugars, serving not only as a carbon source but also as signaling metabolites within the intestinal microenvironment (30). This enables EHEC to modify its proliferative and virulent behavior in response to mucus presence (31). Despite these insights, information on mucus alterations in dogs with EHEC infections is scarce, largely because the detection of mucin protein levels in canines faces significant challenges. This is due to the limited availability of species-specific primary antibodies for mucin. To address this limitation, our study employed SNA staining to detect mucus and utilized a modified fixation process during SEM preparation to enhance the ultrastructural observation of mucus. This context underscores the significance of our findings, which revealed no discernible differences in mucus between infections with non-pathogenic *E. coli* and EHEC (Fig. 6). Such consistency, even with the use of a mucus-preserved fixing method for SEM imaging (Fig. 2B), underscores a robust trend. Further research is essential to fully understand the dynamics of mucus in canine EHEC infections and its role in disease pathogenesis.

In children infected with EHEC, there is a correlation between increased levels of the proinflammatory cytokine IL-8 in the blood and a heightened risk of developing HUS (32). However, this relationship has not been extensively studied in dogs with EHEC infections. In our research, we did not observe a significant rise in IL-8 levels in canine colonoid-derived monolayers infected with EHEC. This absence of a marked increase in IL-8 suggests that the response to EHEC infections in dogs may mirror the generally milder course of EHEC infections seen in dogs under natural conditions. A notable model for studying EHEC infections in humans is the murine model of *Citrobacter rodentium* infection (33, 34). Similar to what we observed in our study, TNF-α levels increase in the gut for this model. Since TNF-α is a known proinflammatory cytokine that regulates macrophage function and these macrophages are protective factors against bacterial infection (35), it seems reasonable that TNF-α expression is induced by EHEC infection *in vivo* in mice and *in vitro* in dogs. However, the expression of TNF-α in mice *in vivo* and in dogs *in vitro* has not been shown. The interaction with immune cells cannot be discussed in this co-culture model of epithelial cells and bacteria alone, so more complex culture systems, such as Gut-on-a-Chip, are needed.

Recent research has explored species-specific variations in EHEC pathogenesis between mice and humans using Gut-on-a-Chip microfluidic technology with human colonoids. This study identified specific microbiome metabolites that contribute to the species-specific susceptibility to EHEC (36). Building on this, we have successfully established a canine Gut-on-a-Chip model using canine intestinal organoids (10). Should we observe parallels in the canine model, especially when comparing intestinal microbiomes from dogs, humans, and potentially mice, this approach could significantly enhance our understanding of microbiome-derived metabolites in EHEC pathogenesis.

Several potential studies and alternative approaches could deepen our understanding of the interactions between EHEC and canine intestinal epithelial cells. Comparative studies involving other O157 isolates (12, 26), as well as stx and/or eae mutants (37–39) in canine colonoid-derived monolayer models, could enrich our understanding. In addition, comparing these findings with those from human colonoid-derived monolayers (12) could further elucidate the mechanistic pathophysiology of EHEC in intestinal epithelial cells.

In conclusion, our study marks a critical step forward in deciphering the complexities of EHEC pathogenesis, with a special focus on canine models. The introduction of a novel canine colonoid-derived monolayer model lays the groundwork for a more nuanced comparative analysis of EHEC strains during infections. Our approach, which includes the strategic modification of culture conditions using saline-only media, successfully limits bacterial overgrowth that typically compromises *in vitro* co-culture experiments. This model, along with a novel canine Gut-on-a-Chip model (10), represents an important opportunity to better understand the role of microbiome metabolites during EHEC infection across different species. The insights gained from such investigations have the potential to guide more effective strategies for the prevention and treatment of EHEC infections, thereby benefiting both animal and human health.

## MATERIALS AND METHODS

### Animals

For this study, three clinically healthy dogs undergoing dental procedures at the Washington State University (WSU) Veterinary Teaching Hospital (VTH) Community Practice Service were included. These dogs, aged between 1 and 12 years, were selected based on comprehensive physical examinations, blood work, and no history of chronic diseases affecting the heart, kidneys, liver, or intestines. Only those dogs deemed fit for elective procedure under general anesthesia were included in the study. This study was conducted with the approval of the Washington State University Institutional Animal Care and Use Committee (IACUC Approval: ASAF#6993), with details on the dogs provided in Table S1.

### Generation of canine colonoids

Colonic stem cells were isolated from the biopsied colonic tissue using the previously reported protocol (8). Briefly, biopsied colonic tissues were cut into small pieces and treated with 30 mM EDTA solution (Invitrogen) for 60 min at 4°C to isolate crypts containing stem cells. These crypts were collected, washed using ice-cold Dulbecco’s phosphate-buffered saline (PBS, Gibco) with 1× penicillin/streptomycin (Gibco), embedded in Matrigel (Corning), and then seeded into 48-well plates (Thermo Scientific). After the Matrigel domes were solidified in a 37°C incubator, 300 µL of organoid culture medium was applied to each well. For the composition of organoid culture medium, we used DMEM/F12 (Gibco) supplemented with 2 mM GlutaMAX (Gibco), 10 mM HEPES (Gibco), 1× penicillin/streptomycin (Gibco), 10% (vol/vol) conditioned medium of Noggin (40), 20% (vol/vol) conditioned medium of R-spondin, 100 ng/mL recombinant murine Wnt-3a (PeproTech), 50 ng/mL murine Epidermal Growth Factor (EGF) (PeproTech), 10 nM gastrin (Sigma-Aldrich), 500 nM A-83-01 (Sigma-Aldrich), 10 µM SB202190 (Sigma-Aldrich), 1 mM N-acetyl-L-cysteine (MP Biomedicals), 10 mM nicotinamide (Sigma-Aldrich), 1× B27 supplement (Gibco), 1× N2 MAX media supplement (R&D Systems), and 100 µg/mL Primocin (Invitrogen). For the initial 2 days following crypt isolation, 10 µM Y-27632 (Stem Cell Technologies) and 2.5 µM CHIR 99021 (Stem Cell Technologies) were added to the culture medium. Once the colonoids were maturated, the Matrigel dome was dissolved using Cell Recovery Solution (Corning), and the colonoids were collected and treated with TrypLE Express (Gibco) and reseeded into 48-well plates.

### Development of canine colonoid-derived monolayers

Once the colonoids reached maturity, the Matrigel dome was dissolved using Cell Recovery Solution (Corning) and colonoids were collected by centrifugation (200 × *g*, 5 min, 4°C). Collected colonoids were treated with TrypLE Express containing 10 µM of Y-27632 for 10 min at 37°C. Subsequently, the dissociated colonoids were filtered through a 70-µm cell strainer (Fisher Scientific) to obtain single cells and the cells were centrifuged at 200 × *g*, 4°C for 5 min.

For the preparation of cell culture inserts (Falcon), these inserts were coated using 100 µg/mL Matrigel and 30 mg/mL collagen I (Gibco) in DMEM/F12 supplemented with 2 mM GlutaMAX, 10 mM HEPES, and 1× penicillin/streptomycin. Dissociated single cells obtained from colonoids were suspended with organoid culture medium supplemented with 10 µM Y-27632 and 2.5 µM CHIR 99021 and seeded onto the pre-coated cell culture insert at a concentration of 1 × 10^6^ cells/mL. The monolayers were cultured in an organoid culture medium containing 10 µM Y-27632 and 2.5 µM CHIR 99021 until the day after seeding, after which the organoid culture medium was changed every other day.

### Microbial culture for infection experiment

This study used an EHEC isolate from cattle to challenge the monolayer and a non-pathogenic *E. coli* strain, MC4100, as an infection control. MC4100 is a derivative of strain K-12, that was originally isolated from a human in 1922 (14) The EHEC isolate was PCR positive for *eae*, *h7, stx1*, and *stx2/vt2* (Fig. S2) (41). Both strains of bacteria were cultured overnight in a sterile Luria-Bertani (LB) medium at 37°C under shaking at 200 rpm. Subsequently, 100 µL of the overnight culture was transferred to 3 mL fresh LB medium and incubated for 3 hours to bring the bacteria to the logarithmic growth phase. After incubation, the bacterial solution was centrifuged at 10,000 x *g* for 3 min, washed with normal saline (0.9% wt/vol NaCl), further centrifuged, and resuspended to 2.0 × 10^7^ CFU/mL in saline.

### Co-culture of bacteria and canine colonoid-derived monolayers

Four days after the start of the culture of the colonoid-derived monolayer, the culture media on both the apical and basolateral chambers were removed and washed three times with saline (Fig. 1A). Saline (200 µL) was added to the apical chamber, and 500 µL of organoid culture medium depleted of antibiotics was added to the basolateral chamber. Then, the colonoid-derived monolayers were further incubated (37°C, 24 h). After incubation, the bacterial suspension was added to the apical chamber to a final concentration of 1.0 × 10^6^ CFU/mL.

### Assessment of intestinal barrier integrity

The intestinal barrier integrity of the colonoid-derived monolayers was evaluated by measuring the TEER value following exposure to bacteria. Electrical resistance (Ω_t_) was quantified using Ag/AgCl electrodes connected to a Volt-Ohm meter (Millicell ERS-2, Millipore), and this value was then transformed into a TEER measurement using the following equation: TEER = (Ω_t_ − Ω_blank_) × A, where Ω_blank_ represents the resistance of the blank well in ohms, and A denotes the surface area of the culture insert in cm^2^.

### Immunocytochemistry of canine colonoid-derived monolayers

Colonoid-derived monolayers were fixed after 36-hour co-culture using 4% PFA (Thermo Scientific), followed by membrane permeabilization with 0.3% Triton-X (Thermo Scientific), blocking with 2% bovine serum albumin (Cytiva), and then the addition of primary antibodies. For visualization of E-cadherin and ZO-1, monoclonal anti-mouse E-cadherin antibody (36/E-cadherin, BD Biosciences) and polyclonal anti-rabbit ZO-1 antibody (61–7300, Invitrogen) were used. SNA staining (Vector Laboratories) was used to visualize goblet cells. CgA staining (ab45179, Abcam) was used to visualize enteroendocrine cells (8, 42). The colonoid-derived monolayers were then washed with PBS and secondary antibodies were added (Anti-Rabbit IgG H&L labeled with Alexa Fluor 555). After further washing, the nuclei and F-actin were visualized using DAPI and Alexa Fluor 647 Phalloidin (Thermo Fisher Scientific), respectively. Then, the monolayer was mounted by Prolong Gold Antifade reagent (Thermo Fisher Scientific) and imaged using a white-light point scanning confocal microscope (SP8-X, Leica). Fluorescence images were acquired under 63× objective, with excitation laser sources of 405 nm, 499 nm, 553 nm, and 653 nm and high-efficiency Leica HyD detector, and processed using LAS X software (Leica). The intensity of the fluorescence signal for E-cadherin and ZO-1 was quantified by performing a line scan using ImageJ 1.54d (43). The mean of the maximum intensity value of each peak was determined by taking the peak intensity value from at least four peaks per line scan and normalizing it by the value obtained from non-pathogenic *E. coli*-infected monolayers. The number of SNA-positive cells per field of view was used to evaluate the number of goblet cells. These evaluations were performed on five randomly selected fields of view from each of the three biological replicates, each of which consisted of three technical replicates.

### Total RNA extraction and qPCR analysis

Total RNA was extracted from a colonoid-derived monolayer utilizing the RNeasy mini kit (Qiagen) and subjected to reverse transcription using the High-Capacity cDNA Reverse Transcription Kit (Applied Biosystems). qPCR was performed using PowerUp SYBR Green Master Mix (Applied Biosystems) and the CFX96 Touch Real-time PCR Detection System (Bio-Rad) to evaluate the gene expression levels of *leucine-rich repeat containing G protein-coupled receptor 5* (*LGR5*), *Chromogranin A* (*CgA*), and *Intestinal Alkaline Phosphatase* (*ALPI*). For cDNA normalization, housekeeping genes were employed, namely, *Succinate dehydrogenase complex subunit A* (*SDHA*), *Hydroxymethyl-bilane synthase* (*HMBS*), and *Hypoxanthine phosphoribosyl-transferase 1* (*HPRT1*), as previously reported in colon tissue (44). Details of the primers used in this study are shown in Table S2.

### Scanning electron microscopy

Monolayers were prepared and processed for SEM using previously established methods (13). Briefly, monolayers were fixed overnight at 4°C using one of two solutions: 2.5% glutaraldehyde in 0.1 M sodium cacodylate buffer (GA) or a combination of 2% glutaraldehyde, 2% paraformaldehyde, and 1.05% alcian blue in 0.1 M cacodylate buffer (GA/PFA/AB). Following the initial fixation, samples were post-fixed with 1% osmium with (GA/PFA/AB) or without (GA) 0.5% tannic acid in 0.1 M sodium cacodylate buffer for 2 hours at room temperature and underwent a dehydration sequence in ethanol concentrations ranging from 30% to 100%, followed by hexamethyldisilazane (HMDS). Samples were coated using a Pt/Pd sputter coater (Cressington High-Resolution Sputter Coater) and imaged with a Quanta 200F SEM (FEI). Bacterial adherence was quantified using SEM on representative images randomly selected from those captured at low magnification (1,542×) of the GA-fixed group. This fixation method was chosen because it does not cover bacteria with mucus, thereby enhancing bacterial visibility and facilitating counting, compared to the GA/PFA/AB fixed group. Ten independent fields of view from three biological replicates were evaluated. The number of bacteria was normalized by the area of one field of view at 1,542× (5,953 µm^2^) and expressed as the number of bacteria per µm^2^.

### Enzyme-linked immunosorbent assay

After the infection experiment was completed, apical and basolateral media were collected. After centrifugation to remove the cellular components of the medium, both media were frozen at −80°C until the inflammatory cytokines were determined by ELISA. The IL-8 Canine ELISA Kit (#ECCXCL8, Invitrogen) and TNF-α Canine ELISA Kit (# ECTNF, Invitrogen) were used to measure IL-8 and TNF-α, respectively, and the assays were performed according to the manufacturer’s protocol. In accordance with previous studies, these measurements were made using basolateral medium (21).

### Statistical analysis

The study was tested in three independent technical replicates using three biological replicates in all analyses. Statistical analysis was conducted using R v4.1.0 (R core team) and figures were generated using GraphPad Prism 10.0.2.232 (Dotmatics). To confirm the normality of each data set, the Shapiro-Wilk test was performed. The Kruskal-Wallis and Dunnett tests were employed for the comparison of TEER value among each condition. The Wilcoxon test was utilized to compare the concentration of IL-8. In addition, Student *t*-tests were applied to compare the number of SNA-positive cells, the fluorescence intensity of tight junction proteins, and the concentration of TNF-α. All results were presented as mean  ±  standard error of the mean (SEM). A *P* < 0.05 was considered statistically significant.

## SUPPLEMENTAL MATERIAL

Figure S1 spectrum.00961-24-s0001.tiff Visualization of Chromogranin A in canine colonoid-derived monolayers using Z-stacking.

Figure S2 spectrum.00961-24-s0002.tiff Characterization of enterohemorrhagic *E. coli*.

Table S1 spectrum.00961-24-s0003.xlsx Signalment of healthy dogs used in this study is summarized.

Table S2 spectrum.00961-24-s0004.xlsx Primer information.

ASM does not own the copyrights to Supplemental Material that may be linked to, or accessed through, an article. The authors have granted ASM a non-exclusive, world-wide license to publish the Supplemental Material files. Please contact the corresponding author directly for reuse.
