# Supplementary figures and images for: Enterohemorrhagic Escherichia coli (EHEC) disrupts intestinal barrier integrity in translational canine stem cell-derived monolayers
Source: Microbiol Spectr. 2024 Aug 20;12(10):e00961-24. doi: 10.1128/spectrum.00961-24 (PMC11448187; doi:10.1128/spectrum.00961-24)

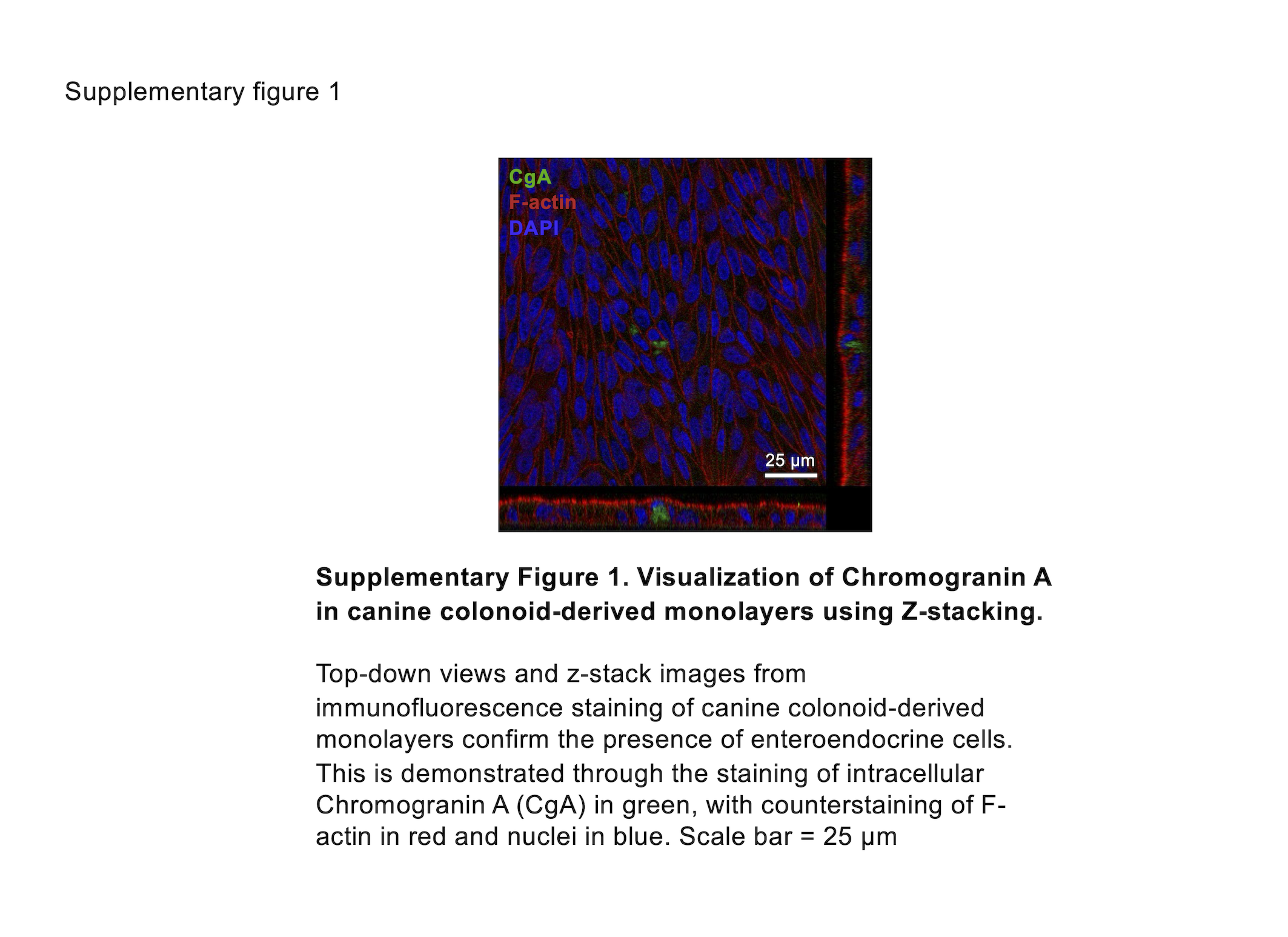

Supplement: Figure S1 — Visualization of Chromogranin A in canine colonoid-derived monolayers using Z-stacking. [file spectrum.00961-24-s0001.tiff]

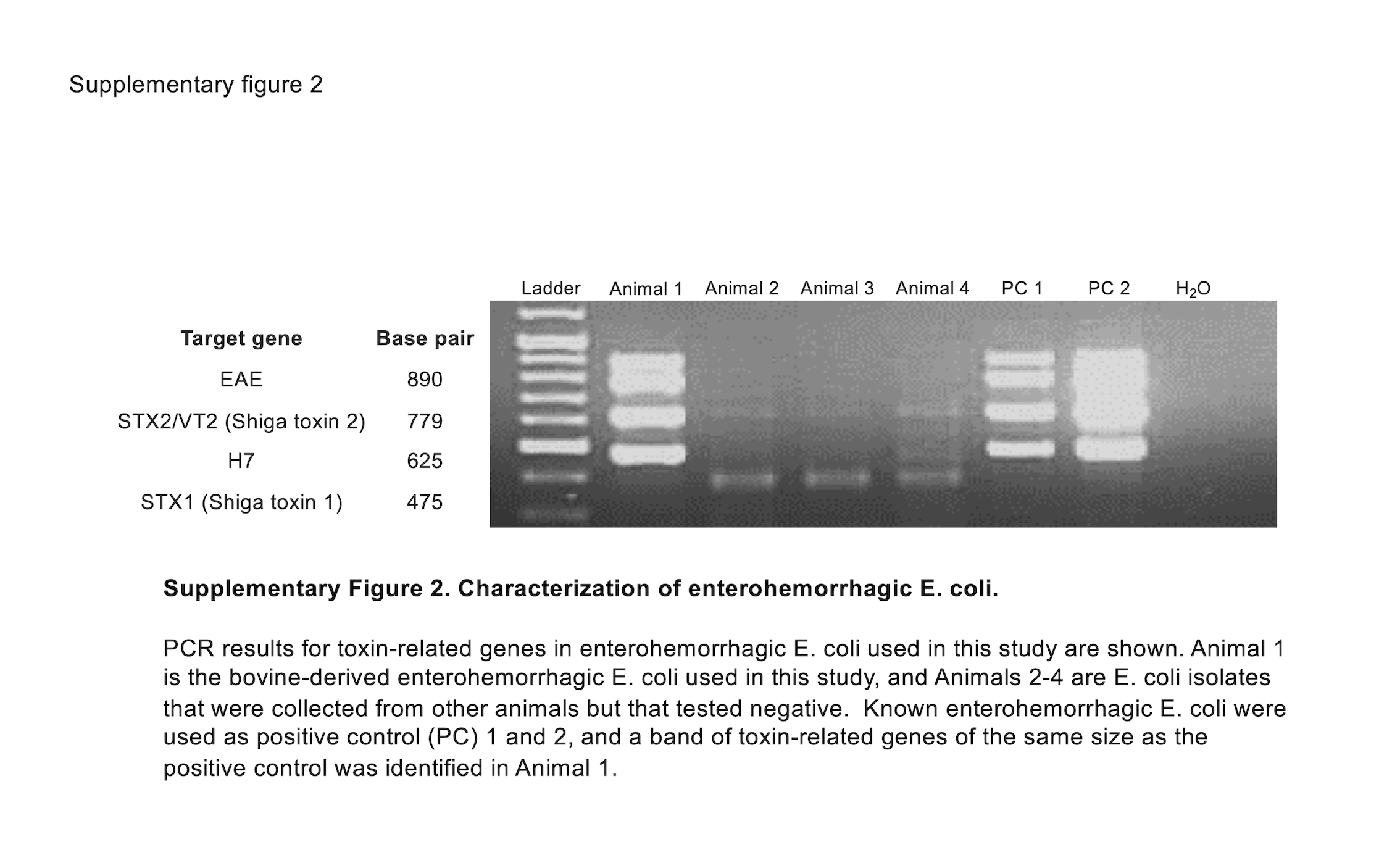

Supplement: Figure S2 — Characterization of enterohemorrhagic E. coli. [file spectrum.00961-24-s0002.tiff]
